# Web-gLV: A Web Based Platform for Lotka-Volterra Based Modeling and Simulation of Microbial Populations

**DOI:** 10.3389/fmicb.2019.00288

**Published:** 2019-02-22

**Authors:** Bhusan K. Kuntal, Chetan Gadgil, Sharmila S. Mande

**Affiliations:** ^1^Bio-Sciences R&D Division, TCS Research, Tata Consultancy Services Ltd., Pune, India; ^2^CSIR-National Chemical Laboratory, Chemical Engineering and Process Development Division, Pune, India; ^3^Academy of Scientific and Innovative Research (AcSIR), Ghaziabad, India; ^4^CSIR-Institute of Genomics and Integrative Biology, New Delhi, India

**Keywords:** microbiome, modeling, numerical-simulation, web-server, time-series, visualization, lotka-volterra, microbial population

## Abstract

The affordability of high throughput DNA sequencing has allowed us to explore the dynamics of microbial populations in various ecosystems. Mathematical modeling and simulation of such microbiome time series data can help in getting better understanding of bacterial communities. In this paper, we present Web-gLV—a GUI based interactive platform for generalized Lotka-Volterra (gLV) based modeling and simulation of microbial populations. The tool can be used to generate the mathematical models with automatic estimation of parameters and use them to predict future trajectories using numerical simulations. We also demonstrate the utility of our tool on few publicly available datasets. The case studies demonstrate the ease with which the current tool can be used by biologists to model bacterial populations and simulate their dynamics to get biological insights. We expect Web-gLV to be a valuable contribution in the field of ecological modeling and metagenomic systems biology.

## Introduction

The ensemble of microbial groups residing in an ecosystem constitutes its microbiome. Mutual interactions between the resident microbes in a given microbiome depend not only on species diversity and abundances, but also on properties of their inhabited environment. On the other hand, the resident microbiota also has a profound influence on the properties of the habitat itself (Levy and Borenstein, 2013; Zelezniak et al., 2015). High throughput sequencing studies, especially for longitudinal microbiome projects, have greatly enhanced our understanding of the nature and dynamics of complex microbial interactions. Temporal analysis of microbial profiles has led to several intriguing findings (Gerber, 2014) and strengthened our understanding of the role of microbes in many diseases. Researchers have also reported new insights such as the existence of multiple steady states in human microbiome using time series microbiome experiments (Gajer et al., 2012; Faust et al., 2015).

Realizing the importance of the dynamic microbiome has encouraged development of methods and tools for its analysis and modeling (Fisher and Mehta, 2014; Bucci et al., 2016; Shaw et al., 2016; Baksi et al., 2018). Some of these tools provide specialized methods to visualize, cluster and compare temporally similar microbial groups, find causal relationships, analyze stationarity, identify community-states, etc. Modeling microbial populations has recently attracted generous attention owing to its capability and potential to forecast future behaviors of the system as well as allow improved estimation of microbial interactions (Berry and Widder, 2014; Fisher and Mehta, 2014). The classical Lotka-Volterra equations can be used to model simple systems such as two species predator prey where the interactions are strictly assumed to be competitive. The “generalized” Lotka-Volterra (gLV) equations on the other hand are an extension of the logistic growth model and are more general than the classical predator-prey (Lotka-Volterra) equations where the interacting species might have a wide range of relationships including competition, cooperation, or neutralism. Such gLVs assume that the interaction (or the effect) of one species with another is encoded in the corresponding coefficient in the equation, providing a powerful framework to model and simulate microbial populations. It must be noted that gLV based models capture the interactions using a single averaged effect in a mean-field type model for which modest computational resource is sufficient. Consequently, it does not account for stochastic fluctuations (random processes), intrinsic dynamic correlations, and cannot address any emerging spatial structures which requires extensive computation. All the caveats applicable to extrapolation of the dynamics of a non-linear system apply to the predictions of the model. However, gLV formulations can still provide a reasonable starting point for more advanced community models and capture the effect of inter microbial associations in a more meaningful way as compared to conventional correlation based methods. Although correlations between groups of microbes can help in revealing underlying ecological processes, they are in most cases insufficient to serve as proxy for microbial interactions (Berry and Widder, 2014; Fisher and Mehta, 2014). Parameter estimations using Lotka-Volterra based models have been demonstrated to be better than correlation based measures (Fisher and Mehta, 2014). Additionally, the gLV models can provide an estimate of the native growth rates of uncultured microbes. While a positive value of the “interaction coefficient” is assumed to be a beneficial effect, a negative value indicates an inhibitory effect. If the coefficient has a zero value, no interaction is assumed to be present between the two taxa. The gLV equations were first used to model the interaction between bacteria and yeast in a cheese microbiome (Mounier et al., 2008) and thereafter in a few more microbiome studies (Marino et al., 2014; Dam et al., 2016; Vos et al., 2017; Venturelli et al., 2018). Simulation studies using generalized Lotka-Volterra (gLV) models can be used to understand microbiome dynamics and can assist biologists to design better experimental strategies. For a given microbial community (with known abundance and diversity), gLV can also be used to predict the future state of the microbiome. Similarly, it can be utilized to understand the temporal behavior of the microbiome if the initial conditions are perturbed.

Tools like LIMITS (Fisher and Mehta, 2014), MetaMis (Shaw et al., 2016), and MDSINE (Bucci et al., 2016) are available for applying gLV modeling on microbial time series data. LIMITS and MetaMis focus mainly on reconstruction of microbial interactions and are available as Mathematica code and an offline Matlab based GUI, respectively. MDSINE, although providing the most comprehensive suite of functionalities for analysis, requires knowledge of Matlab programming. In this communication, we present a web based tool called “Web-gLV” (freely available at http://web.rniapps.net/webglv) which can be used for modeling, visualization, and analysis of microbial populations without any programming expertise and has no installation requirements (Supplementary Table 1). Users can either upload a microbial time series abundance data matrix to formulate the mathematical models automatically or can provide pre-calculated model parameters, namely the growth rate, and inter-microbial interaction matrix. The outcomes of the simulations can be used to obtain various biological insights and enable optimization of experimental designs. “Web-gLV” is expected to be a valuable addition to the suite of tools in the field of ecological modeling and metagenomic systems biology.

## Results

“Web-gLV” provides an easy platform for biologists to exploit the benefits of gLV modeling by simply uploading the experimentally obtained time series microbial abundance data. The application is flexible to allow users input microbial growth rates and interaction values if known from other sources. We demonstrate the utility of “Web-gLV” using few publicly available datasets.

### Case-Study 1: Predicting the Future State of Gut Microbiome

In this simulation, we used an available longitudinal metagenomic time series data of gut microbiome samples from a healthy human subject (Caporaso et al., 2011). The aim of this case study was to model the temporal behavior of top five dominant microbial taxa present in healthy human gut microbiome and use the model for predicting temporal dynamics of a future state which is unknown to the model. In order to achieve this, we used the above dataset to create a gLV model using the first 100 time points and considered the 101th time point as a start point to predict the abundance profiles of the subsequent 30 time points. The predicted 30 time points were then compared with the experimentally reported abundance profiles (Supplementary Figure 1A). In order to evaluate how close “Web-gLV” predicted trends are with respect to the experimentally observed trends, a Dynamic Time Warping (DTW) based algorithm (Berndt and Clifford, 1994) was used. DTW can evaluate the similarity between two time series of equal or unequal lengths using a dynamic programming based approach and can be used to successfully capture equivalence in the overall pattern. The low DTW distances between the observed and predicted trajectories (Supplementary Figure 1B) indicated that the gLV model was able to capture the observed temporal patterns in the selected taxonomic groups with good accuracy. The predicted dynamics could capture *Lachnospira'*s positive influence on *Faecalibacterium* as well as its negative influence on *Akkermansia, Bacteroides*, and *Phascolarctobacterium* (Supplementary Figure 1C). Cyclic trends in the *Bacteroides* abundance (as prevalent in the observed trends) were also seen to be well captured in the predicted trajectories. In order to evaluate the robustness of the predicted trends, we changed the initial abundance values (to half and one fourth) of the two most abundant taxa, namely *Bacteroides*, and *Akkermansia*. With these changes, the predicted abundances of the two taxa did not show much deviation in their temporal trends (Supplementary Figure 2). Therefore, as expected, these two taxa, being the most abundant, were seen to be robust to different initial values. To check the similarities in the trends of the selected taxa over time, DTW distance metric was used to generate the dendograms (Supplementary Figure 1D). The obtained results indicated a good agreement between the observed and “Web-gLV” predicted trees, thereby validating the simulation capability of gLV models. The details of the individual steps followed in the case study are explained under the “Methods” section.

### Case-Study 2: Understanding Changes in Microbial Interaction Patterns Upon Perturbation

The ability of gLV modeling to decipher interaction patterns in a microbial community was exploited in this case study to find differences between a healthy and perturbed gut microbiome. In order to understand the effect of perturbation on the dominant microbial genera, we used the publicly available time series microbiome data corresponding to *Clostridium difficile* infection (Bucci et al., 2016). The dataset consisted of regularly sampled time-series microbiome abundances (for 28 days) in five gnotobiotic mice pre-colonized with human commensal bacterial strains which were later infected with *C. difficile* spores. The data also included measured microbial abundances for additional 28 days post infection in these mice. For constructing the gLV model and predicting microbial interactions in the unperturbed state of the microbiome, we used the top five abundant taxa from data corresponding to the pre *C. difficile* infection time points of all five mice samples. Similarly, in order to construct a representative model and predict microbial interactions in the perturbed state, we considered the post *C. difficile* infection time points. Thus, two models, namely “normal state model” and “perturbed state model”, were generated for each mouse sample using gLV modeling implemented in “Web-gLV.” A biological realistic constraint enforcing positive intrinsic growth and negative or zero self interaction (Bucci et al., 2016) was considered during model generation (see Methods for details).

The predicted interaction profiles revealed a clear difference in the nature of microbial interactions between “normal” and “perturbed” states for all the five samples (Supplementary Figure 3). Upon inspecting the changes in the nature of interactions of individual taxa (from their normal to perturbed state) across all the samples, it was observed that genera exhibiting the maximum change differed in each of the samples (Supplementary Figure 3). For example, while *Akkermansia muciniphila* showed the least change across a majority of the mice samples (“Mouse 1,” “Mouse 3” and “Mouse 4”), no clear cut pattern was observed for other taxa. Overall, the total negative interactions decreased in most of the samples (“Mouse 3,” “Mouse 4” and “Mouse 5”) but every mouse displayed a unique combination of interaction profiles. This may be explained as an effort by the dominant players (taxa) in the microbiome, each trying in its own way to influence the individual sample level variations.

In order to evaluate whether the model generated using the perturbed state of one mouse is able to predict the perturbation dynamics of the other mice, we predicted the post perturbation trajectories corresponding to each mouse, considering perturbation model of every other mice. Results of this simulation indicated an overall good prediction of the temporal dynamics (Supplementary Figure 4).The results corresponding to the predicted perturbed state trajectories of a mouse based on the model of its own normal state is shown (Supplementary Figure 4). To achieve this, gLV models were also generated for the normal states corresponding to all the mice samples (see Methods for details). In the next step, we checked whether these predictions could be improved by incorporating the normal state of the same mouse in combination with the perturbed state of another mouse. The comparison of the predicted and observed perturbation dynamics for each of the subjects was performed by evaluating their sum DTW distances. Comparison of the results indicated that the perturbed state of a mouse could be predicted better using the perturbed state of another mouse instead of using the model corresponding to the normal state of the same mouse. Interestingly, utilizing the normal state model of the same mouse in combination with the perturbed state model of another mouse did not show a consistent improvement (Supplementary Figure 4). Thus, the results indicate that the growth rate and interaction parameters of perturbation dynamics are better encoded in a comparable perturbation model rather than the normal state model of the same subject. However, additional advanced modeling steps like inclusion of antibiotic susceptibilities are expected to further enhance prediction accuracies (Bucci et al., 2016). The main objective of this case study was to demonstrate the capability of “Web-GLV” to use growth rate and interaction parameters derived from other experiments to perform simulations on new data in a user friendly way.

## Conclusions

The increased affordability of DNA sequencing has enabled researchers to move beyond the hypothesis generated using static snapshots of microbiome. Lotka-Volterra based modeling provides an efficient means to leverage the current volume of generated longitudinal microbiome data. The generalized Lotka-Volterra (gLV) modeling extends the classical two species predator prey models which are widely used in ecology. An important advantage of gLV models is its ability to estimate the native growth and interaction parameters of uncultured microbes in a given environment from temporal data which would otherwise be difficult using traditional culture based methods (Bucci and Xavier, 2014). Consequently, using these parameters, one can study the changes in microbial communities over time starting with unknown initial conditions. This allows testing of new hypothesis and helps to gather improved mechanistic insights. However, the intricacies of the advanced mathematical concepts involved as well as their implementation might prove to be a hindrance for many biologists. The limited availability of “ready-to-use” tools for such analysis also serves as a bottleneck to quickly test a hypothesis and obtain meaningful insights. We have developed “Web-gLV” to bridge this gap and to enable biologists take advantage of the multispecies modeling and simulation without any programming expertise. “Web-gLV” also bypasses any installation needs and requires only the time series microbial abundance data as input. A set of interactive operations allows easy initialization and simulation of microbial population as well as analysis of the output trajectories. Results of repeated simulations can be easily evaluated by altering the initial values and the parameters using GUI based inputs. The interactive graphical plots generated by the tool aids in easy analysis and comparison of the results. We demonstrate the ease with which Web-gLV can be used to automatically model and simulate microbial communities and generate outputs. Furthermore, we demonstrate the accuracy of the predictions and possible biological interpretations of the results.

Although gLV based models provide a good starting point for modeling microbial community dynamics, it does not account for random processes which forms essential part of any biological system. Additionally, with the increase in number of species and time span of prediction, the simulation output is also prone to numerical errors. Consequently, Web-gLV limits simulating a maximum of 10 species at a time for at the most 100 time points. The compositionality bias in microbiome data arising due to sampling and sequencing limitations may also cause inaccurate estimation of simulation parameters. Moreover, too much irregularity in the sampled time points may also result in inaccurate parameter estimations. Hence, it is advised to cautiously interpret the findings obtained using Web-gLV and more importantly augment it with the underlying biology of the systems (Faust and Raes, 2012; Gerber, 2014).

## MaterialS, Methods, and Implementation

### Modeling and Parameter Estimation of a Generalized Lotka-Volterra Equation

A multi species gLV model for rate of change of a counts “*x*_*i*_” of a species “*i*” can be written as an ordinary differential equation as shown below:

(1) $$\begin{array}{r} \frac{dx_{i}}{dt}=x_{i}\left(r_{i}+\;\overset{n}{\underset{j=1}{\sum }}\propto_{ij}x_{j}\right)\ldots \end{array}$$

where, *r*_*i*_ corresponds to the intrinsic specific growth rate of species “*i*” and ∝_*ij*_ is the influence on the growth rate of species “*i*” exerted by another species “*j*” of the community consisting of “*n*” species. Thus, for a given set of “*n*” species, “*n*” differential equations can be formulated which can then be used for simulating the behavior of those species starting with a set of initial values. However, in order to perform such simulation, one also needs to find the values of other types of parameters for each of the equations namely the growth rate *r*_*i*_ and the set of inter-species interaction parameter ∝_*ij*_.

The Equation (1) can be rewritten as below:

(2) $$\begin{array}{r} \frac{1}{x_{i}}\frac{dx_{i}}{dt}=\left({\text{r}}_{\text{i}}+\;\overset{\text{n}}{\underset{\text{j}=1}{\sum }}\propto_{\text{i}\text{j}}{\text{x}}_{\text{j}}\right)\;\ldots \end{array}$$

Further, Equation (2) can be expressed as:

(3) $$\frac{d\ln(x_{i}\left(t\right))}{dt}=\;\left(r_{i}+\;\sum_{j=1}^{n}\propto_{ij}x_{j}\right)\ldots$$

For numerical integration following the implicit trapezoid method, upon discretizing Equation (3) for each sub interval (let [*k*,*k*+1]), and taking the average value of *x*_*j*_ we get:

(4) $$\begin{array}{l} \ln x_{i}\;\left(\;t_{k+1}\right)-\;\ln x_{i}\;\left(\;t_{k}\right)\\ \;\approx \;\left(r_{i}+\;\overset{n}{\underset{j=1}{\sum }}\propto_{ij}\left\{\frac{\left({x_{j}}_{\left(k+1\right)}+\;{x_{j}}_{k}\right)}{2}\right\}\right)\Delta t\;\ldots \end{array}$$

**Figure 1:**
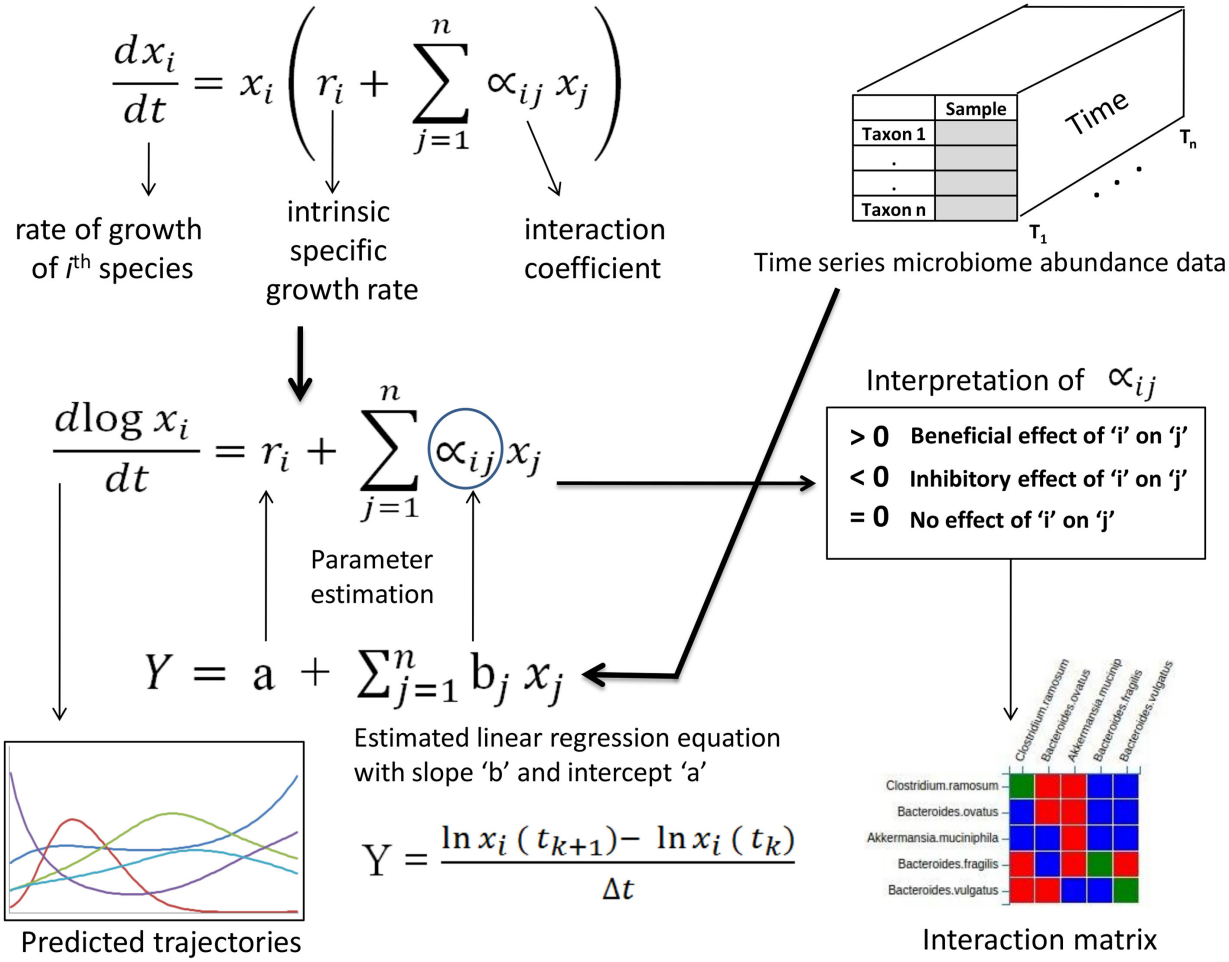
Overview of formulation and use of multi species generalized Lotka-Volterra (gLV) models for obtaining microbial interactions and predict future trajectories.

Now, given a time series data for abundances of the set of “*n*” species, these two parameters namely *r*_*i*_ and ∝_*ij*_ can be estimated by comparing equation (4) to a linear regression model for log lagged differences in abundances estimated for each *i*^th^ taxa (*x*_*i*_) available in the microbiome time series data wherein the intercept corresponds to the *r*_*i*_ values and the coefficients to the ∝_*ij*_ values (Figure 1). Earlier studies have suggested using a constrained regression (with enforced positive intrinsic growth and negative or zero self interaction constraints) for microbial populations as it is biologically more realistic (Bucci et al., 2016). Web-gLV implements two methods for parameter estimation namely PLSR (Partial least squares regression) for unconstrained estimation (Haenlein and Kaplan, 2004) and LSEI algorithm (Haskell and Hanson, 1978) for constrained estimations (Supplementary Figure 5). The constrained estimation solves a least square problem under conditions where *r*_*i*_ is forced to take a positive value and ∝_*ii*_ values are constrained to less than or equal to zero.

### Evaluation of Predicted Trajectory

The observed and predicted trajectories are compared using a Dynamic Time Warping algorithm (DTW). DTW measures the similarity between two time series (with or without a lag) using a dynamic programming approach (Berndt and Clifford, 1994) and can be used to compare time series of unequal lengths. As, in most cases, the compared trajectories in “Web-gLV” are expected to be unequal, DTW fits as the best scoring metric. If “T1” and “T2” are two time series vectors of length “*m*” and “*n*,” respectively, DTW finds a mapping path {(*p*_1_*,q*_1_),(*p*_2_*,q*_2_),…,(*p*_*k*_*,q*_*k*_)} with boundary conditions (*p1,q1*) = (1,1) and (*p*_*k*_*,q*_*k*_) = (*m,n*). The DTW distance between T1 and T2 for a point (*i, j*) is calculated by solving a dynamic programming using the distance formula shown below:

(5) $$\begin{array}{r} DTW\left(i,j\right)=|T1\left(i\right)-\;T2\left(j\right)|+min\left\{\begin{array}{c} DTW\left(i-1\right),j\\ DTW\left(i-1,j-1\right)\\ DTW\left(i,j-1\right) \end{array}\right\}\ldots \end{array}$$

To calculate the final distance, a matrix MDTW of dimensions m×n is constructed after filling MDTW(1,1) with the initial condition value of MDTW(1,1) = |*T*1(1) − *T*2(1)|. The whole matrix is then filled one element at a time using the formula shown in Equation 5. The final distance value is available at the cell MDTW(*m,n*). The distance is calculated between the scaled (between 0 and 1) time series belonging to the “Observed” and “Predicted” data which is presented as a table along with the trend plots in the “Web-gLV” tool. The sum total (or cumulative) DTW distance for a set of predicted taxa can be used as a measure to score the similarity between two or more simulations. Additionally, the “all vs. all” DTW distance is calculated for the “Observed” and “Predicted” data to generate the hierarchically clustered dendograms. These dendograms can be useful to understand the microbial community structure.

### Implementation of the Web-gLV Tool

**Figure 2:**
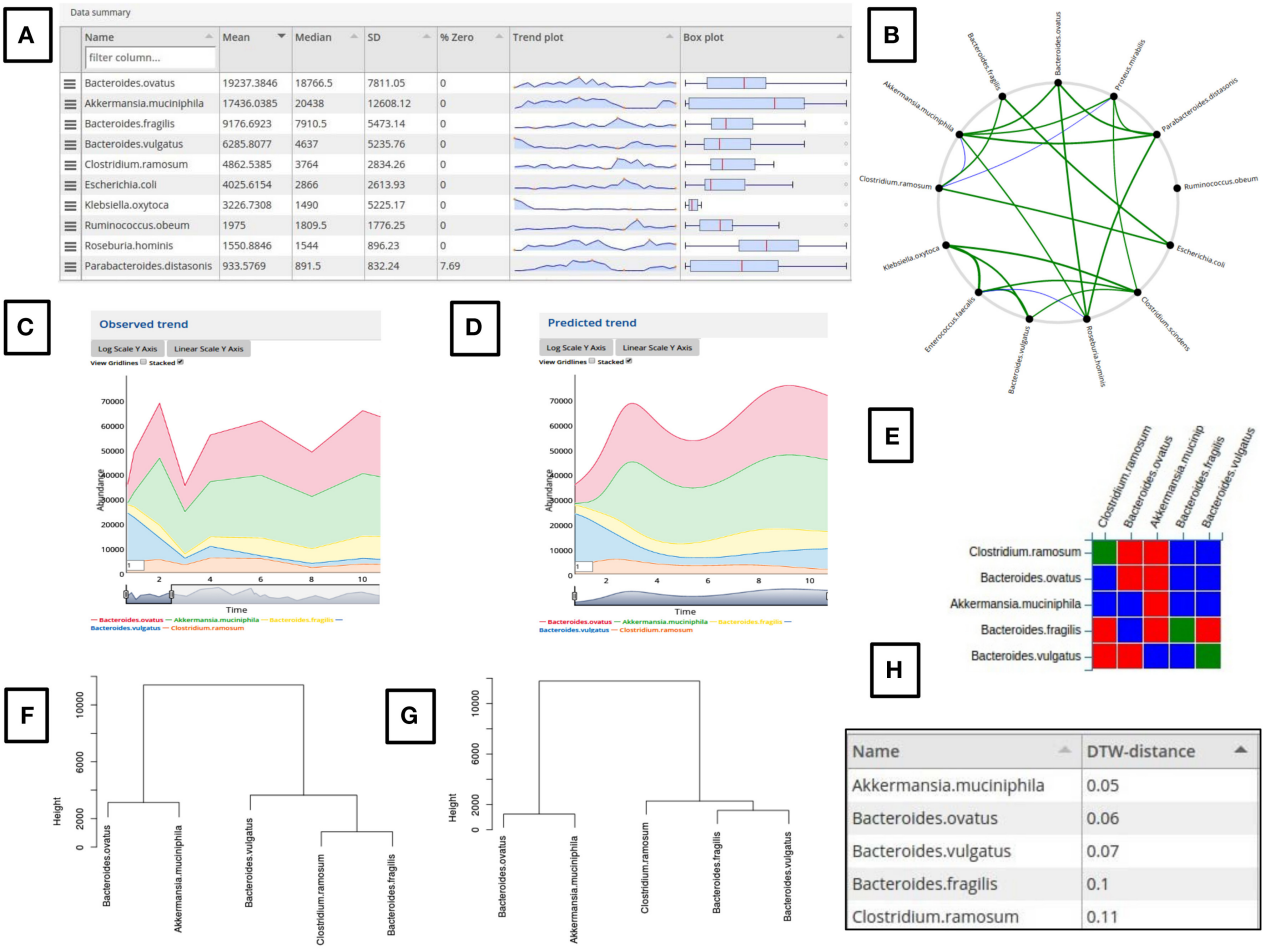
Demonstration of the various features of the web-gLV tool. **(A)** Tabulated summary of the input microbiome abundance data. **(B)** Microbial association network generated from the input data which can be used to select the required taxa to be used for modeling. **(C)** A stacked line plot based comparison of the observed **(C)** and predicted **(D)** trajectories. **(E)** A matrix representation of the predicted interaction coefficients for the modeled taxa (Red, Negative, Blue, Positive and Green, No interaction). Dendogram based comparison of the change in the microbial community structure between the observed **(F)** and the predicted **(G)** trends. **(H)** Evaluation of the similarity between the observed and predicted time series curves scored using a DTW metric.

Web-gLV has been developed using JavaScript (and PHP) for the frontend with R (deSolve package) and Perl scripts in the backend (Soetaert et al., 2010). The tool can perform simulations starting two types of input sets. A user can either upload only a taxonomic abundance file which will be used to estimate parameters and generate reference plots for the observed trends. Alternatively, in addition to a taxonomic abundance file, a growth rate file and inter-taxa interaction file can also be uploaded separately to bypass the automatic parameter estimation step and use the supplied values for numerical simulation. A metadata file corresponding to the timepoints specified in the main taxonomic abundance file can also be uploaded as an optional input. This metadata information will be used by the tool to augment the time series plots based on the available information. The reference values of initial starting point of simulation for the selected taxa set can be selected from one of the time point row of this abundance table. Once the input files are uploaded, the various steps involved in running a simulation are described below:

**Step 1**: Selecting the taxa required for simulation from the input dataset:

Given a time series microbiome data as input, the tool presents a tabulated graphical summary in the form of box plots, trend charts and other accompanying statistics of the input microbial abundance profiles (Figure 2A). Additionally, a Pearson correlation (*r* ≥ 0.5 and *r* ≤ −0.5) based network is created using the core taxa (having <30% zeroes in the sampled longitudinal timescale) (Figure 2B). This network can be viewed by clicking on the link “Click here to show/hide correlation network.” The taxonomic groups desired to be added for modeling can be selected using the graphical summary table, the dropdown search box or the correlation network. Clicking on a taxa label in the summary table adds that taxa to the simulation. Similarly, clicking on a node in the network adds it and the connected nodes. This feature can be used to select a set of closely related microbial groups showing a correlated temporal behavior. Adding a taxa for simulation using the above two methods also makes it visible in the searchable dropdown along with a graphical display of its temporal behavior in the “Observed trend” window. This dropdown can also be used to remove added taxa or add more taxa by selecting from the dropdown. Adding or deleting a taxon automatically updates the “Observed trend” plot. Several user interactive operations like log transformation, stacking/un-stacking, viewing gridlines and selecting a desired window of the trend plot is possible. A moving average based smoothing can also be applied to the time series plot by modifying the value in the left bottom corner box (Figures 2C,D).

**Step 2**: Selecting simulation parameters:

After selecting the taxonomic groups, a user has to specify the modeling parameters like start and end point of data time-points for estimating the interaction coefficients, numerical simulation interval duration and the solver used for numerical integration of the ordinary differential equations (ODE) method. The interaction coefficients for the equation are then inferred using a partial least square regression (if selected for unconstrained growth rate selection) or a constrained regression (if selected for an enforced positive intrinsic growth and negative/zero self interaction constraints). Other parameter estimation methods that require numerical integration at each step of the optimization process are potentially better in terms of accuracy but require substantially more resources and time than the implemented methods. Earlier studies have suggested using the constrained method for modeling microbial populations as it is biologically more realistic (Bucci et al., 2016). The start time (or initial value) for the simulation can be interactively selected as any one of the time-point from the input dataset with provision to edit the values. This option can be used to test perturbations in the initial microbial abundance values and observe the simulation outcomes. It needs to be noted that the “Parameter estimation” settings are not available when a simulation is started with a user supplied growth rate and interaction file.

**Step 3a**: Running the simulation:

After setting the parameters, The “Run simulation” button can be clicked to perform a simulation. If the simulation is successful, the predicted trajectories for the selected taxa are displayed under the “Predicted trend” window (Figure 2D). The observed vs. predicted trend plot for a taxon is also generated as a mixed plot with the observed trends shown in points connected by dotted lines and the predicted as firm lines of same color. In case of an unsuccessful simulation due to incorrect parameter or solver limitation, an error message is displayed and no trajectories are generated. The timeseries plot in the “Observed trend” window is automatically set to display the selected time range if the simulation range matches. This feature is helpful to compare the predicted trajectories from a modified starting point and compare it with the unmodified observed trends. The predicted growth rate and interaction coefficient matrix (Figure 2E) which was used for simulation is displayed graphically for convenience. A simulation can be re-run by altering some parameter/simulation settings as well as with a modified set of initial values.

**Step 3b**: Performing cross predictions:

“Web-gLV” can also be used to perform cross predictions by estimating growth and interaction parameters in one simulation and use the same to predict dynamics in a different simulation. The predicted parameters can be saved as text files using the “Download table” option available under “Predicted Intrinsic Growth Rates” and “Predicted Interaction Matrix” headers in the “Web-gLV” tool. While performing a new simulation with a similar type of taxonomic groups whose time series abundances are available, the downloaded parameters can be uploaded to perform the simulation. This feature available in the “Web-gLV” tool can be used to test the prediction performance of models on unknown initial conditions as demonstrated in case study 2.

**Step 4**: Evaluating the simulation output:

The predicted trajectories are scored for their similarities (Figure 2H) with the observed time series using a Dynamic Time Warping (DTW) distance metric (Berndt and Clifford, 1994). The all vs. all DTW metric is used to construct a hierarchal clustered dendogram for the observed and predicted trends (Figures 2F,G). These dendograms represents the temporal similarities between the selected microbial groups and hence a reflection of their community structure. A comparison of the dendograms generated for the “Observed” and “Predicted” data can hence be used as a measure of the simulation prediction accuracy.

### Numerical Validation of Web-gLV Predictions

Web-gLV implements two methods for parameter estimation namely PLSR (Partial least squares regression) for unconstrained estimation (Haenlein and Kaplan, 2004) and LSEI algorithm (Haskell and Hanson, 1978) for constrained estimations. We used standard R modules namely pls and limSolve, respectively, for the backend implementation. The tool is designed to capture trends, which provides an idea of the growth rate and nature of interactions. However, for an improved understanding, it is imperative to look into the functional potential of the participating taxonomic groups (Nagpal et al., 2016; Bhatt et al., 2018). Web-gLV can provide a good starting point for more advanced community models by augmenting information from other sources. We compared both the constrained as well as unconstrained parameters estimated by web-gLV with previously reported methods as demonstrated in section introduction of Data Sheet 1. It should be noted that the calculated coefficients for the constraint optimization solves the same problem in different ways providing non-unique solutions. Consequently, the parameters are free to take any values depending on the solution which may result in differences between the estimated parameter values. However, as expected, the predicted trajectories (when evaluated for the case studies) show a good agreement between the various tools (Section results of Data Sheet 1).

### Using “Web-gLV” to Perform the Case Studies

The modeling and simulations involved in the case studies demonstrated in the “Results” section were performed completely using the “Web-gLV” tool. The datasets are available in the home page of the tool which can be auto-loaded by selecting the “View” button corresponding to each case study. The first 100 time point for case study 1 were selected using Timepoint 1 (sampling interval: 0) as start and Timepoint 100 (sampling interval: 143) as end under the “parameter estimation settings.” The future 30 time points were predicted by selecting Timepoint 101 (sampling interval: 144) as start and setting the “Time duration” option to 30 under “simulation settings.” For case study 2, the start and end time points for creating the “normal” state models corresponded to Timepoint 1 (sampling interval: 0.75) and Timepoint 13 (sampling interval: 28), respectively. Similarly, for the perturbed models, Timepoint 1 (sampling interval: 28.75) and Timepoint 26 (sampling interval: 56) corresponded to the start and end time points, respectively. The solver for numerical simulation was selected as ODE45 for both the case studies with time interval as 0.1. A biological realistic constraint enforcing positive intrinsic growth and negative or zero self interaction was applied for generating all the modes by selecting the option under “parameter estimation settings.” However, the constrained parameter optimization failed to find an exact solution for the “normal” state model of “Mouse 5” for which we unselected the option and generated the model without the constraints.

## Data Availability

The link https://web.rniapps.net/webglv contains the data-sets used in the case study along with the user manual for running the Web-gLV tool.

## Author Contributions

BK conceived the idea, implemented the algorithms and developed the interface. BK, CG, and SM designed the case studies, evaluated the results, and drafted the manuscript. All authors read and approved the final manuscript.

### Conflict of Interest Statement

BK and SM are employed by the company Tata Consultancy Services Limited. The remaining author declares that the research was conducted in the absence of any commercial or financial relationships that could be construed as a potential conflict of interest.
