# Corrigendum: Web-gLV: A Web Based Platform for Lotka-Volterra Based Modeling and Simulation of Microbial Populations

**DOI:** 10.3389/fmicb.2020.605308

**Published:** 2021-01-08

**Authors:** Bhusan K. Kuntal, Chetan Gadgil, Sharmila S. Mande

**Affiliations:** ^1^Bio-Sciences R&D Division, TCS Research, Tata Consultancy Services Ltd., Pune, India; ^2^CSIR-National Chemical Laboratory, Chemical Engineering and Process Development Division, Pune, India; ^3^Academy of Scientific and Innovative Research (AcSIR), Ghaziabad, India; ^4^CSIR-Institute of Genomics and Integrative Biology, New Delhi, India

**Keywords:** microbiome, modeling, numerical-simulation, web-server, time-series, visualization, lotka-volterra, microbial population

In the published article, there was an error in affiliation 3. Instead of “CSIR-National Chemical Laboratory, Academy of Scientific and Innovative Research, Pune, India,” it should be “Academy of Scientific and Innovative Research (AcSIR), Ghaziabad, India”.

The authors apologize for this error and state that this does not change the scientific conclusions of the article in any way. The original article has been updated.

