# Supplementary material for: Web-gLV: A Web Based Platform for Lotka-Volterra Based Modeling and Simulation of Microbial Populations
Source: Front Microbiol. 2019 Feb 22;10:288. doi: 10.3389/fmicb.2019.00288 (PMC6394339; doi:10.3389/fmicb.2019.00288)
Supplement: Supplementary file 1 [file Data_Sheet_1.PDF]

Supplementary material 1

1. Validation of predicted parameters :

Web-gLV implements two methods for parameter estimation namely PLSR (Partial least squares regression) for unconstrained estimation and LSEI algorithm (as described by Lawson and Hanson 1974, 1995) for constrained estimations. We used standard R modules namely pls and limSolve respectively for the backend implementation. The tool is designed to capture trends, which provides an idea of the nature of the interactions. However, for an improved understanding of microbial interaction, it is imperative to look into the functional potential of the participating taxonomic groups. Web-gLV provides a good starting point for more advanced community models.

1.1 Comparison of parameters calculated using unconstrained estimation (PLSR methodology):

In order to validate the PLSR estimated parameters by web-gLV, we used a publicly available dataset (Kloppers and Greeff, 2013) where the authors applied gLV to simulate the percentage of the market share captured by the three competitors 'x', 'y' and 'z' respectively using a set of observations from historical data. In the original study, the authors provided an analytical solution to estimate the gLV parameters using the log integral method. We uploaded the same dataset into the web-gLV application and noted the predicted parameters. The unconstraint estimation option was chosen for this case as it uses a PLSR (partial least square regression). The predicted parameters agreed completely with the ones reported by the authors as shown in Table 1 below (also shown along with the predicted trends in Supplementary Figure 5):

		Log integral method	PLSR in web-gLV
Competitor X	**intercept**	4.3432	4.343169808
	**coeff_x**	-4.4069	-4.406853867
	**coeff_y**	-4.0595	-4.05951412
	**coeff_z**	-4.727	-4.726983605
Competitor Y	**intercept**	0.5734	0.573391611
	**coeff_x**	0.329	0.329793334
	**coeff_y**	-1.5848	-1.584783594
	**coeff_z**	-0.3285	-0.328493453
Competitor Z	**intercept**	-3.617	-3.616956857
	**coeff_x**	3.4259	3.425911167
	**coeff_y**	3.8169	3.816920652
	**coeff_z**	3.5911	3.591122206

Table 1: Comparison of gLV parameters using PLSR (in web-gLV) with the Log integral method

1.2 Comparison of parameters calculated using constrained estimation (LSEI algorithm):

In order to validate the interaction parameters predicted by web-gLV under constrained estimation (using the LSEI algorithm), we compared our results with the one implemented in the MDSINE package (maximum likelihood constrained ridge regression: MLCRR). The MLCRR methods may be potentially better in terms of accuracy but requires substantially more resource and time making it difficult for a web-server deployment. The MetaMis tool was also included for comparative evaluation purpose although it uses a different algorithm for parameter estimation. We used the same dataset of our case study 2 (Mouse 1) for predicting the interaction parameters using all the time points in MDSINE, web-gLV and MetaMis. As the growth rate predictions were not available in MetaMis tool, we could only use the ones predicted by MDISNE. The results showed an agreement between most of the growth parameters and about half of the predicted interaction parameters as shown in Table 2 and Table 3 below. It should also be noted that the predicted coefficients (growth rate and interaction coefficients) for the methods solves a constraint optimization problem in different ways providing non-unique solutions. Consequently, the parameters are also free to take any values depending on the solution.

Microbial taxa	MDSINE	web-gLV
Akkermansia-muciniphila	1.6634	3.198610985
Bacteroides-fragilis	0.9587	1.641432825
Bacteroides-ovatus	0.81413	0.101028643
Bacteroides-vulgatus	0.080829	0.001
Clostridium-ramosum	0.44948	0.465364211

Table 2: Comparison of predicted growth rates parameters in web-gLV with MDSINE

Taxa 1	Taxa 2	Metamis	MDSINE	web-gLV
Bacteroides-ovatus	Akkermansia-muciniphila	7.10E-05	8.82E-06	3.08E-05
Bacteroides-ovatus	Bacteroides-fragilis	-3.68E-05	5.11E-06	-3.18E-05
Bacteroides-ovatus	Bacteroides-vulgatus	-1.14E-05	5.96E-05	2.14E-05
Bacteroides-ovatus	Clostridium-ramosum	-3.73E-05	-5.42E-05	-4.50E-05
Akkermansia-muciniphila	Bacteroides-ovatus	-9.23E-06	-9.73E-06	-6.36E-06
Akkermansia-muciniphila	Bacteroides-fragilis	-2.88E-06	-7.21E-05	-8.51E-06
Akkermansia-muciniphila	Bacteroides-vulgatus	-1.40E-05	0.000194	-2.55E-05
Akkermansia-muciniphila	Clostridium-ramosum	-3.41E-06	-0.00028	1.05E-05
Bacteroides-fragilis	Bacteroides-ovatus	-1.33E-05	-2.98E-05	-7.36E-06
Bacteroides-fragilis	Akkermansia-muciniphila	-0.00016	1.69E-06	-7.83E-05
Bacteroides-fragilis	Bacteroides-vulgatus	1.77E-05	6.11E-07	1.16E-05
Bacteroides-fragilis	Clostridium-ramosum	7.48E-05	-6.99E-05	7.68E-05
Bacteroides-vulgatus	Bacteroides-ovatus	-1.14E-05	-1.02E-06	1.90E-05
Bacteroides-vulgatus	Akkermansia-muciniphila	-4.83E-05	-9.80E-06	6.80E-05
Bacteroides-vulgatus	Bacteroides-fragilis	-3.89E-05	-1.64E-05	-3.30E-05
Bacteroides-vulgatus	Clostridium-ramosum	1.68E-05	0.000137	2.81E-05
Clostridium-ramosum	Bacteroides-ovatus	-4.67E-05	-4.69E-06	1.75E-06
Clostridium-ramosum	Akkermansia-muciniphila	-0.00028	-2.20E-05	-0.00043
Clostridium-ramosum	Bacteroides-fragilis	-8.90E-05	8.23E-05	-0.00013
Clostridium-ramosum	Bacteroides-vulgatus	8.04E-05	4.43E-05	3.84E-05

Table 3: Comparison of predicted interaction parameters between MetaMis, MDSINE and web-gLV. Consensus predictions based on the nature of interaction (positive or negative) are highlighted in light green.

2. Validation of predicted trajectories :

Once the parameters of a gLV equation are estimated, it can be used to simulate the trajectories of a time stretch given an initial start point. These predictions are performed by using numerical integration to solve the ODE (Ordinary Differential Equation) generated by the gLV equation. Several methods are available for obtaining this numerical solution and allow specifying the time duration and interval for the simulation. However, with the increase in time duration as well as the step size, the predictions are prone to accumulate numerical errors. We used the datasets for both the case studies (referred in the manuscript) to generating gLV models using constraint estimation and evaluated the trajectories for each taxon using a RMSD (Root Mean Square Distance) calculated as:

$$\text{RMSD (O, P)} = \sqrt{\frac{\sum_{t=1}^{T}(O_t - P_t)^2}{T}}$$

Where O_t and P_t represents the observed and predicted abundance of the taxon at time ‘t’ evaluated for a total ‘T’ time points. In order to compare the predictions, we used one of the most popular solver (ODE 45) implemented in web-gLV tool (using the R deSolve package) and selected a small time step of 0.1 and a prediction time stretch of 20 for all the simulations. The initial points were selected as Timepoint 101 (sampling interval: 144) and Timepoint 13 (sampling interval: 30) for case-study 1 and case-study 2 dataset respectively. The results of the simulations showed a good agreement between the MDSINE and web-gLV predictions as shown in Figure 1 and Figure 2 below.

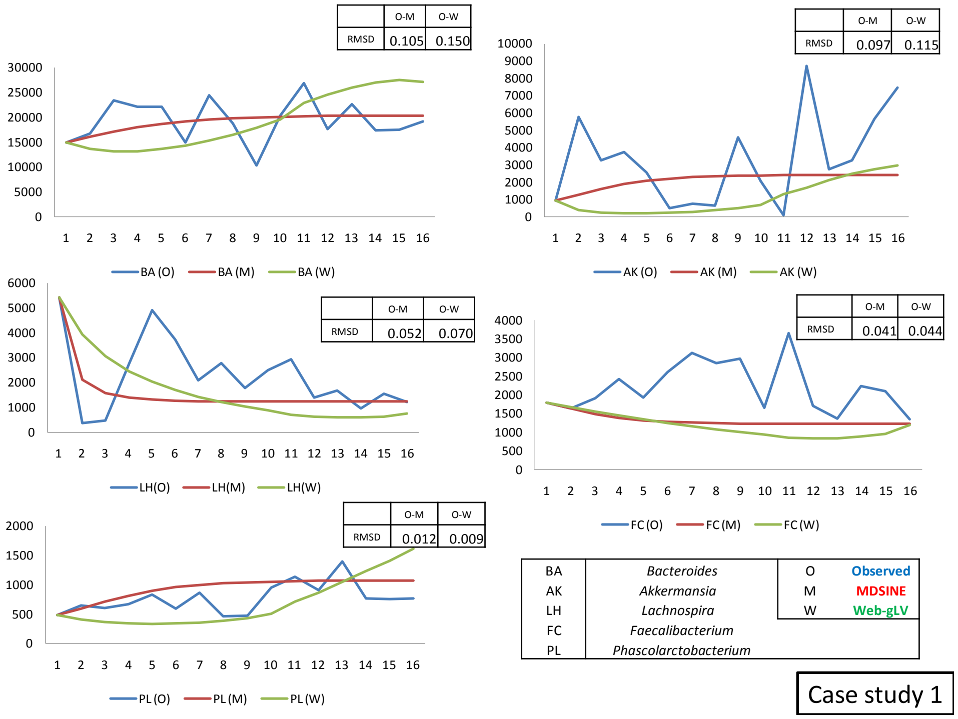

Figure 1: Comparison of simulated trajectories between MDSINE and web-gLV for case study 1

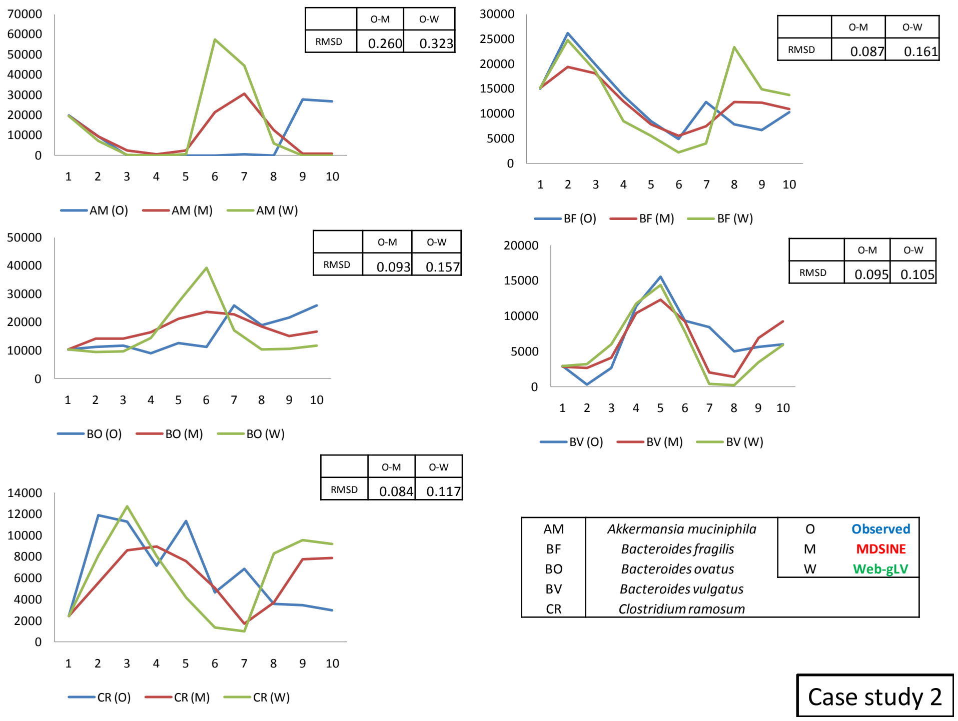

Figure 2: Comparison of simulated trajectories between MDSINE and web-gLV for case study 2

As the MetaMis tool did not allow exporting the predicted trajectory outputs, we could not compare the individual trajectories. The simulated trajectories for MetaMis could only be captured as images along with a provided cumulative Bray-Curtis dissimilarity (BCD) score. Consequently, we evaluated our predictions results with MetaMis using only the BCD score calculated as:

$$BCD\ (O_t, P_t) = \sqrt{\frac{\sum_{i=1}^{M} |O_{it} - P_{it}|}{\sum_{i=1}^{M} (O_{it} + P_{it})}}$$

Where O_{it} and P_{it} represents the observed and predicted abundance of the taxon 'i' at time 't' evaluated for 'M' taxa. The cumulated BCD is calculated by summation of all the BCD values for 'T' matched time points.

Case study 1 | Case study 2

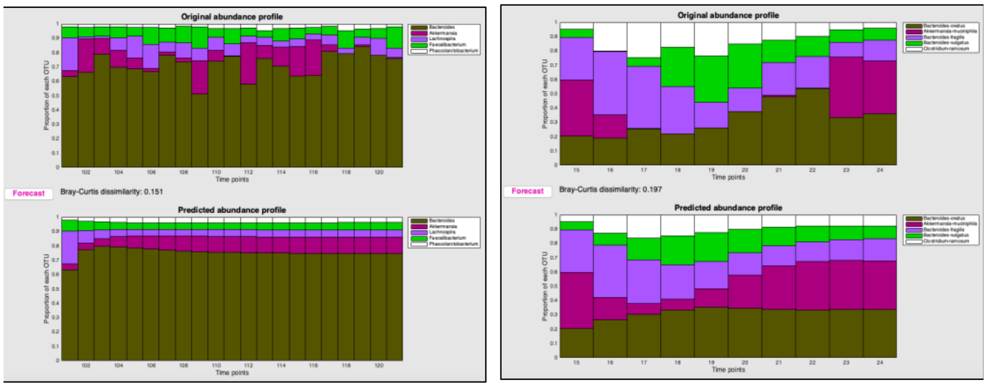

**MetaMis**	0.151
**MDSINE**	0.116
**Web-gLV**	0.151

**MetaMis**	0.197
**MDSINE**	0.245
**Web-gLV**	0.315

Tables represent the Bray-curtis dissimilarity compared for all the predictions

Figure 3: Comparison of simulated trajectories between MetaMis, MDSINE and web-gLV for the two case studies using BCD
