# Supplementary material for: Web-gLV: A Web Based Platform for Lotka-Volterra Based Modeling and Simulation of Microbial Populations
Source: Front Microbiol. 2019 Feb 22;10:288. doi: 10.3389/fmicb.2019.00288 (PMC6394339; doi:10.3389/fmicb.2019.00288)
Supplement: Supplementary file 2 [file Table_1.DOCX]

**Supplementary table 1**

|  | **LIMITS**  learning Interactions from Microbial Time Series Data | **MetaMis**  Metagenomic Microbial Interaction Simulator | **MDSINE**  Microbial Dynamical Systems Inference Engine | **Web-gLV**  Web server for generalized Lotka-Volterra simulation |
| --- | --- | --- | --- | --- |
| **Dependency** | Mathematica | MATLAB (Requires Matlab runtime environment to be installed) | MATLAB | No dependency (only browser required) |
| **Usage complexity** | High (Mathematica Programming expertise required) | Low (Graphical user interface) | High (MATLAB Programming expertise required) | Low (Graphical user interface) |
| **Parameter estimation method** | Sparse Linear Regression | Partial Least Square Regression | Maximum-likelihood constrained ridge regression and Bayesian algorithms | Partial Least Square Regression |
| **Support for Constrained parameter estimation** | No | No | Yes | Yes |
| **Platform** | Standalone for Mac, Windows, Linux | Standalone for Mac and Windows (64 bit) | Standalone for Windows, Mac and Linux | Web server for any platform |
| **Visualization of results** | No | Inbuilt visualizations (limited interactivity) | Visualization can be performed using the accompanied R-scripts to generate static plots | Interactive visualizations |
| **Availability** | Available as a supplementary material of the original publication | <https://sourceforge.net/projects/metamis> | <https://bitbucket.org/MDSINE/mdsine/> | https://web.rniapps.net/webglv/ |

**Unique features of Web-gLV tool :**

1. Allows easy uploading user defined parameters (growth rates and inter microbial interaction matrix) and perform simulations.
2. Comprehensive visual summary (using box and trend plots) of the input microbial abundance data which can be interactively used for selecting the taxa intended for simulation. Additionally, Web-gLV generates a microbial association network, which can also be used for selecting the starting taxonomic groups.
3. A fully interactive trajectory viewer for visualizing the input time series as well as compare the predicted results. Users can selectively view a part of the trajectory for a selected set of microbes and apply a log transformation on the abundance values. The view (for the observed plot) is automatically set to the simulation time range for easy comparison. In addition, the viewer also allows performing a moving average based smoothing on the displayed trends using an input box (provided at the left bottom corner).
4. Starting point of simulation (‘seed’) can be interactively selected from the input dataset and can be easily modified to induce known perturbations (by editing the starting point values).
5. A Dynamic time warping (DTW) distance based comparison of observed and predicted trends and a tree based comparison to assess the changes in microbial community after a simulation.
6. Flexible selection and modification of parameters to easily perform different simulations.
