# Supplementary material for: Web-gLV: A Web Based Platform for Lotka-Volterra Based Modeling and Simulation of Microbial Populations
Source: Front Microbiol. 2019 Feb 22;10:288. doi: 10.3389/fmicb.2019.00288 (PMC6394339; doi:10.3389/fmicb.2019.00288)
Supplement: Supplementary file 3 [file Presentation_1.pptx]

## Slide 1
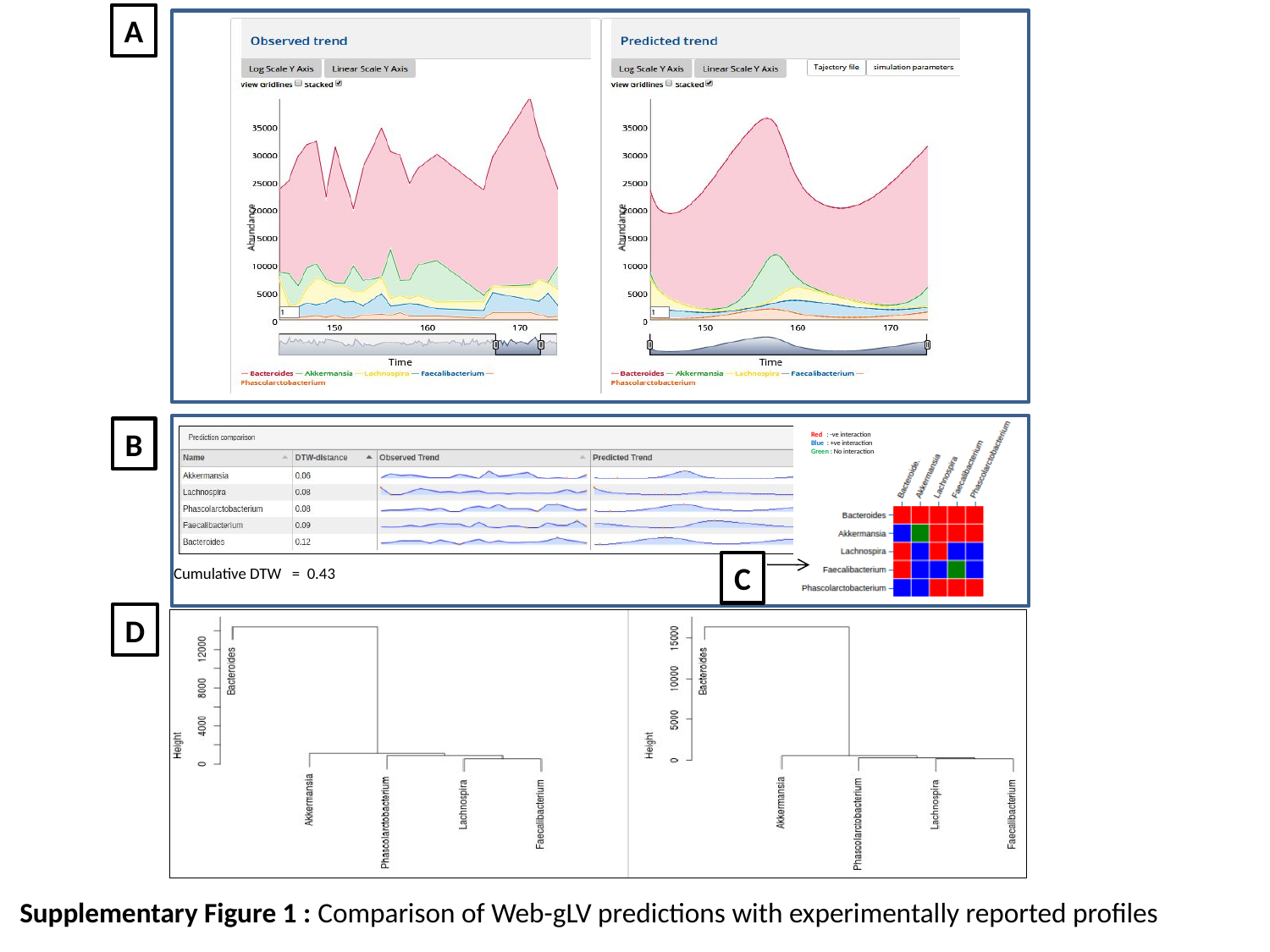

A
B
Red : -ve interaction
Blue : +ve interaction
Green : No interaction
C
Cumulative DTW = 0.43
D
Supplementary Figure 1 : Comparison of Web-gLV predictions with experimentally reported profiles

## Slide 2
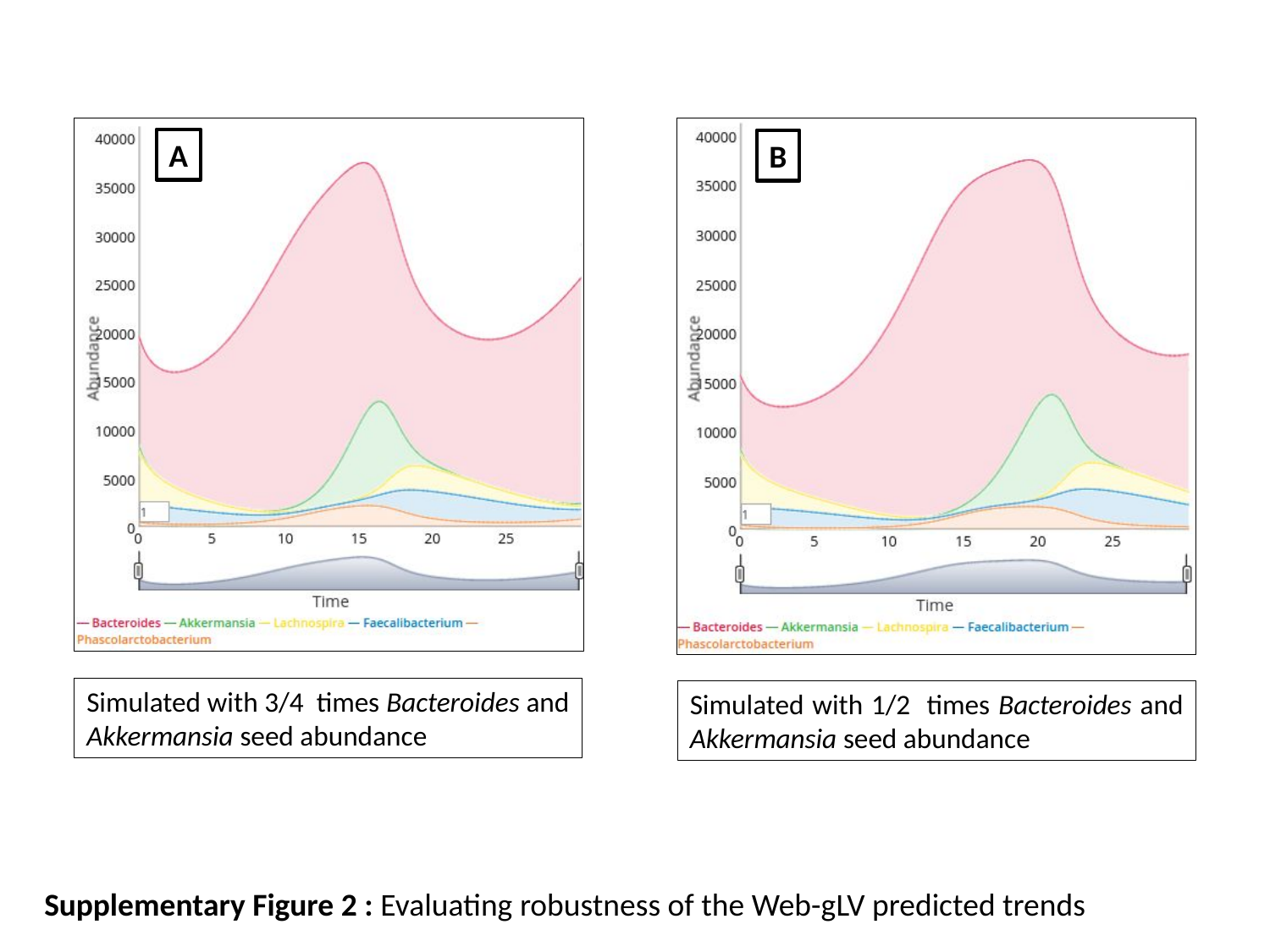

A
B
Simulated with 3/4 times Bacteroides and Akkermansia seed abundance
Simulated with 1/2 times Bacteroides and Akkermansia seed abundance
Supplementary Figure 2 : Evaluating robustness of the Web-gLV predicted trends

## Slide 3
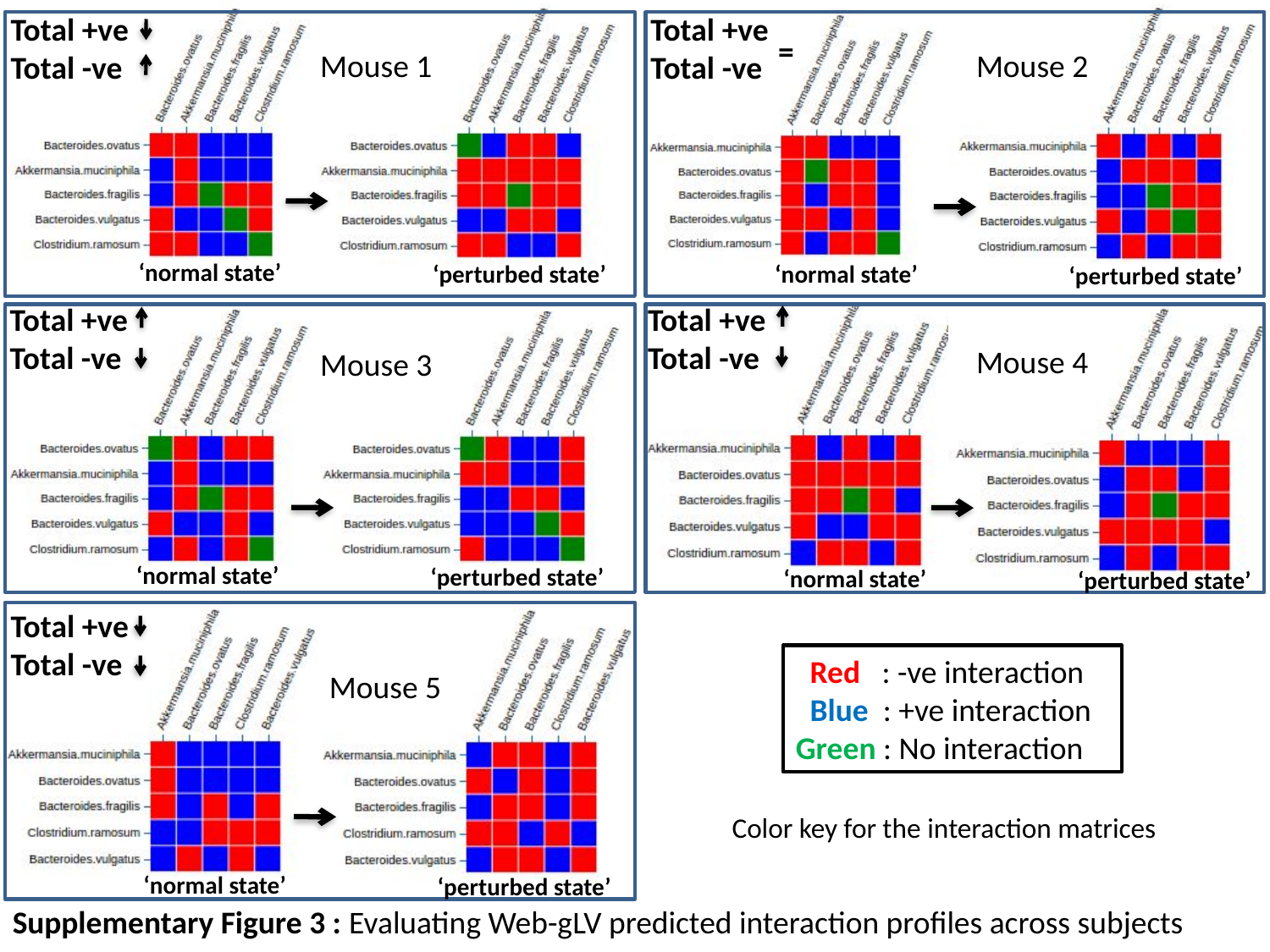

Total +ve
Total -ve
Total +ve
Total -ve
=
Mouse 1
Mouse 2
‘normal state’
‘perturbed state’
‘normal state’
‘perturbed state’
Total +ve
Total -ve
Total +ve
Total -ve
Mouse 4
Mouse 3
‘normal state’
‘perturbed state’
‘normal state’
‘perturbed state’
Total +ve
Total -ve
 Red : -ve interaction
 Blue : +ve interaction
Green : No interaction
Mouse 5
Color key for the interaction matrices
‘normal state’
‘perturbed state’
Supplementary Figure 3 : Evaluating Web-gLV predicted interaction profiles across subjects

## Slide 4
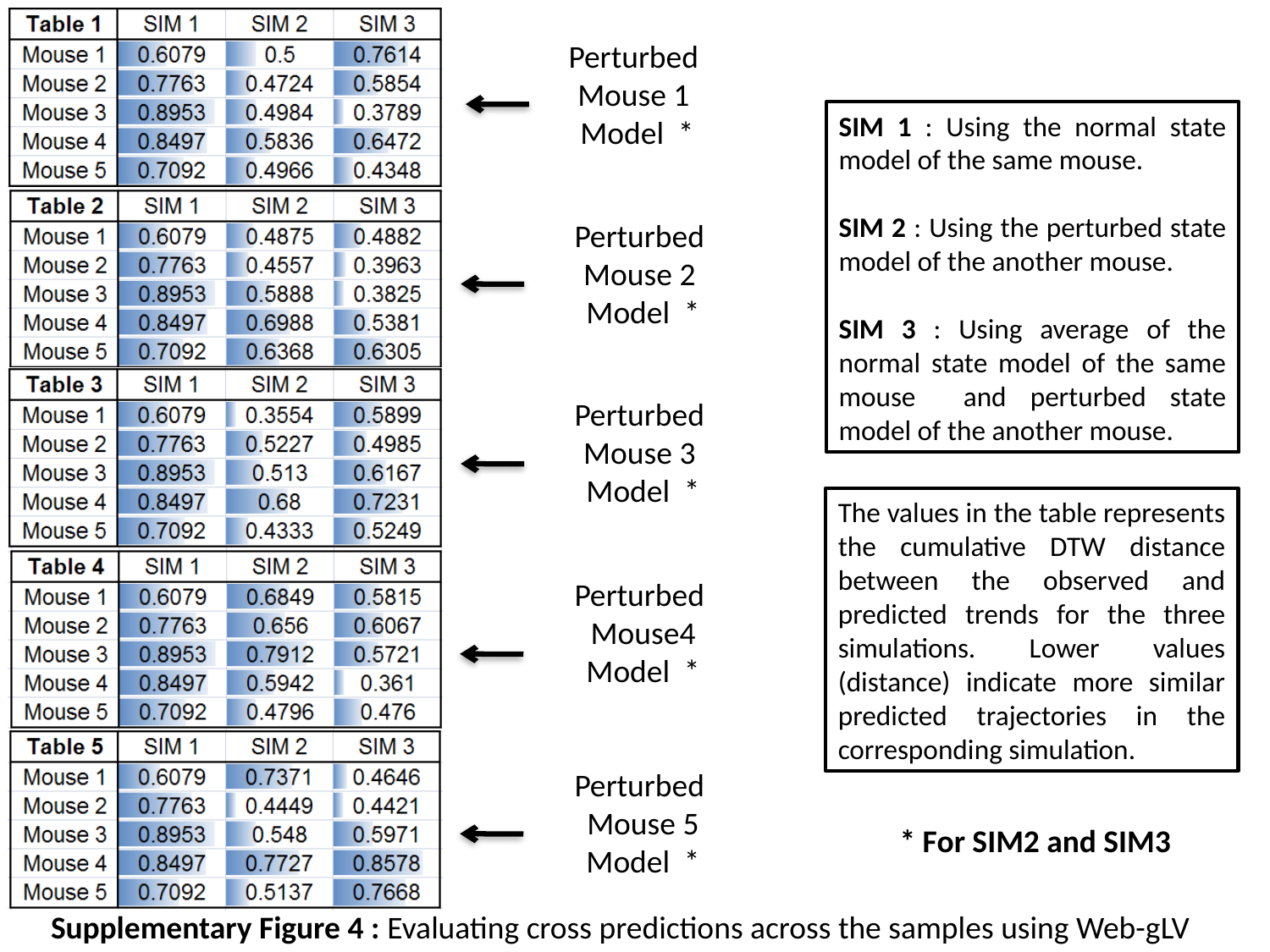

Perturbed
Mouse 1
Model *
SIM 1 : Using the normal state model of the same mouse.
SIM 2 : Using the perturbed state model of the another mouse.
SIM 3 : Using average of the normal state model of the same mouse and perturbed state model of the another mouse.
Perturbed
Mouse 2
Model *
Perturbed
Mouse 3
Model *
The values in the table represents the cumulative DTW distance between the observed and predicted trends for the three simulations. Lower values (distance) indicate more similar predicted trajectories in the corresponding simulation.
Perturbed
Mouse4
Model *
Perturbed
Mouse 5
Model *
 * For SIM2 and SIM3
Supplementary Figure 4 : Evaluating cross predictions across the samples using Web-gLV

## Slide 5
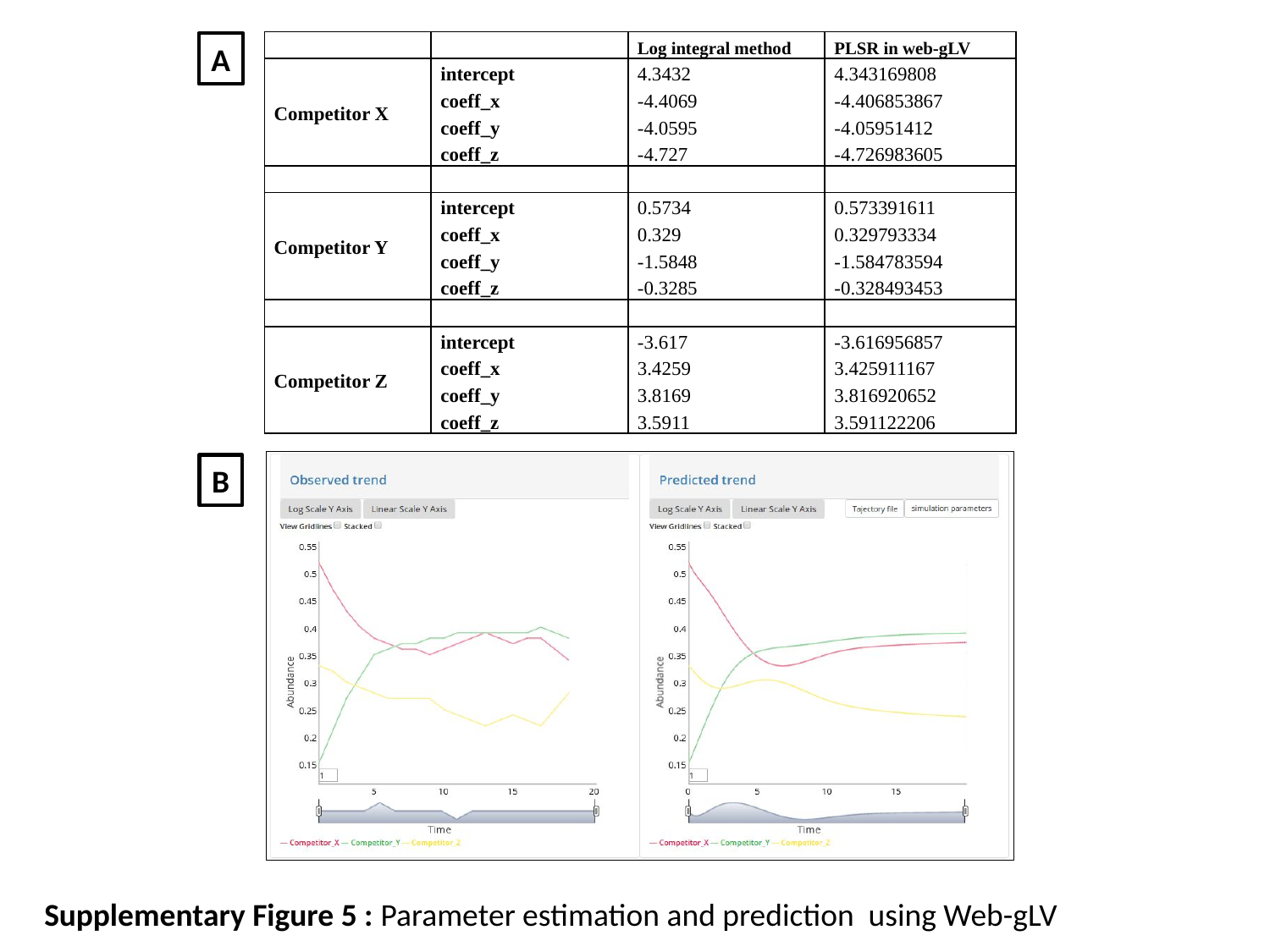

| | | Log integral method | PLSR in web-gLV |
| --- | --- | --- | --- |
| Competitor X | intercept | 4.3432 | 4.343169808 |
| | coeff\_x | -4.4069 | -4.406853867 |
| | coeff\_y | -4.0595 | -4.05951412 |
| | coeff\_z | -4.727 | -4.726983605 |
| | | | |
| Competitor Y | intercept | 0.5734 | 0.573391611 |
| | coeff\_x | 0.329 | 0.329793334 |
| | coeff\_y | -1.5848 | -1.584783594 |
| | coeff\_z | -0.3285 | -0.328493453 |
| | | | |
| Competitor Z | intercept | -3.617 | -3.616956857 |
| | coeff\_x | 3.4259 | 3.425911167 |
| | coeff\_y | 3.8169 | 3.816920652 |
| | coeff\_z | 3.5911 | 3.591122206 |
A
B
Supplementary Figure 5 : Parameter estimation and prediction using Web-gLV
